# The pathogenicity scoring system for mitochondrial tRNA mutations revisited

**DOI:** 10.1002/mgg3.47

**Published:** 2013-11-11

**Authors:** Emiliano González-Vioque, Belén Bornstein, María Esther Gallardo, Miguel Ángel Fernández-Moreno, Rafael Garesse

**Affiliations:** 1 Departamento de Bioquímica, Instituto de Investigaciones Biomédicas “Alberto Sols” UAM-CSIC and Centro de Investigación Biomédica en Red de Enfermedades Raras (CIBERER), Universidad Autónoma de Madrid28029, Madrid, Spain; 2 Laboratorio de Enfermedades Mitocondriales, Instituto de Investigación Sanitaria Hospital 12 de Octubre (i+12)Madrid, Spain; 3 Servicio de Bioquímica, Instituto de Investigación Sanitaria Puerta de Hierro MajadahondaMadrid, Spain

**Keywords:** Mitochondrial tRNA, *MT-TK*, pathogenicity, scoring system, *trans*-mitochondrial cybrid study

## Abstract

Confirming the pathogenicity of mitochondrial tRNA point mutations is one of the classical challenges in the field of mitochondrial medicine. In addition to genetic and functional studies, the evaluation of a genetic change using a pathogenicity scoring system is extremely useful to discriminate between disease-causing mutations from neutral polymorphisms. The pathogenicity scoring system is very robust for confirming pathogenicity, especially of mutations that show impaired activity in functional studies. However, mutations giving normal results using the same functional approaches are disregarded, and this compromises the power of the system to rule out pathogenicity. We propose to include a new criterion in the pathogenicity scoring systems regarding mutations which fail to show any mitochondrial defect in functional studies. To evaluate this proposal we characterized two mutations, m.8296A>G and m.8347A>G, in the mitochondrial tRNA^L^^ys^ gene (*MT-TK*) using *trans*-mitochondrial cybrid analysis. m.8347A>G mutation severely impairs oxidative phosphorylation, suggesting that it is highly pathogenic. By contrast, the behavior of cybrids homoplasmic for the m.8296A>G mutation is similar to cybrids containing wild-type mitochondrial DNA (mtDNA). The results indicate that including not only positive but also negative outcomes of functional studies in the scoring system is critical for facilitating the diagnosis of this complex group of diseases.

## Introduction

Mitochondria are the main source of cellular ATP through the activity of the oxidative phosphorylation (OXPHOS) system. OXPHOS system is composed of five different complexes embedded in the inner mitochondrial membrane (complexes I–V) and two small electron carriers, namely ubiquinone and cytochrome *c*. It contains subunits encoded in the nuclear and the mitochondrial genomes which are physically separated (Garesse and Vallejo 2001). Mitochondrial DNA (mtDNA) encodes a limited number of genes essential for OXPHOS biogenesis, including two rRNAs (12S and 16S) and 22 tRNAs, along with 13 proteins that correspond to respiratory chain subunits (Garesse and Vallejo 2001). The tRNAs encoded in the mitochondrial genome are the only ones used in protein biosynthesis within this organelle; the use of only 22 tRNAs in mitochondrial protein synthesis, in contrast, for example to the 45 employed by *Escherichia coli*, illustrates the low redundancy of human mitochondrial genetics (Wittenhagen and Kelley 2003).

Mitochondrial DNA is especially susceptible to accumulating point mutations. Many of these mutations have moderate or no phenotypic effects, but others cause severe diseases (Schapira 2012). Mitochondrial encephalomyopathies caused by mutations in mtDNA are a heterogeneous group of disorders characterized by primary dysfunction of the OXPHOS system. This OXPHOS dysfunction causes defective mitochondrial ATP synthesis which affects predominantly high energy-demanding tissues such as the nervous system and skeletal and cardiac muscles. Since 1988, when the first pathogenesis-associated mutations in the mitochondrial genome were described (Holt et al. 1988; Wallace et al. 1988), over 250 mutations associated with diseases have been identified within the human mitochondrial genome. More than half of these disease-related mutations are located within tRNA genes, underscoring the essential role of mitochondrial tRNAs in the synthesis of proteins involved in energy metabolism (Zifa et al. 2007). However, the pathogenic mechanism related to most of these disease-causing mutations is still unknown. Furthermore, and because of the marked variability in mtDNA, it is often difficult to discriminate between a pathogenic mutation and a neutral polymorphism (Montoya et al. 2008). Until now, different approaches including genetic, biochemical, and cellular studies have been extensively used to help in the correct classification of mtDNA mutations and in the characterization of their molecular and biochemical phenotype. One of these approaches, based on the classical canonical criterion of pathogenicity described by DiMauro and Schon (2001), is the pathogenicity scoring system developed by McFarland et al. (2004), which was recently reevaluated in order to increase its sensitivity for classifying mutations as pathogenic (Yarham et al. 2011).

This scoring system gives a special weight to functional data, such as those obtained from *trans*-mitochondrial cybrid or single-fiber studies, which are considered the gold standard methods for assigning pathogenicity. First described in 1989 (King and Attardi 1989), the *trans-*mitochondrial hybrid (cybrid) model is one of the most useful tools for studying the consequences of mtDNA defects on cellular metabolism and, therefore, identifying pathogenic mtDNA mutations (Trounce and Pinkert 2007). Notwithstanding the usefulness of the information obtained from cybrid studies, only results confirming an OXPHOS defect are taken into account when mutations are studied using this approach, while studies showing normal mitochondrial function are not taken into consideration. With the aim of showing the usefulness of considering normal behavior in functional studies to discriminate between a pathogenic mutation and a neutral polymorphism, the effect of two different nucleotide changes in the *MT-TK* gene (m.8296A>G and m.8347A>G) were characterized. These nucleotide changes are associated with several mitochondrial diseases, but their pathogenicity remains controversial. On the basis of the results obtained, we proposed a modification in the widely used pathogenicity scoring system of Yarham et al. (2011).

## Methods

### Cell lines and media

Two myoblast cell lines harboring the m.8296A>G or the m.8347A>G mtDNA nucleotide changes, and a myoblast cell line harboring wild-type mtDNA, were kindly obtained from Professor Yuichi Goto. Myoblast cell lines were grown in DMEM-F12 1:1 supplemented with 10% fetal bovine serum (FBS) and 50 *μ*g/mL uridine. The human osteosarcoma cell line 143 B (TK^−^) was grown in DMEM supplemented with 10% FBS, 100 *μ*g/mL bromodeoxyuridine and 50 *μ*g/mL uridine. Its mtDNA-less derivative (143B/206-*ρ*0) was grown in DMEM supplemented with 5% FBS and 50 *μ*g/mL uridine.

For all assays 1 × 10^6^ cells were plated on 10 cm^2^ dishes 48 h before the experiment in 10 mL of DMEM containing 2 mg/mL glucose, 2.5 mg/mL galactose, 110 *μ*g/mL pyruvate, 50 *μ*g/mL uridine and 10% FBS.

### Establishment of *trans*-mitochondrial hybrids

Patients’ derived enucleated myoblasts (0.5 × 10^6^ cells) were fused with 10^6^ *ρ*^0^-206 cells as described previously (King and Attadi 1996). Cybrids were grown in DMEM supplemented with 10% dialyzed FBS and 100 *μ*g/mL bromodeoxyuridine. To rule out major nuclear influences and to avoid clonal effects, we generated and studied at least two independent clones per mutation. In all clones, the mtDNA was sequenced completely to confirm the presence of the mutations and to rule out non polymorphic sequence variations. In addition, restriction fragment length polymorphism analysis has been carried out to check that in all cases the mtDNAs were in homoplasmy.

### mtDNA sequencing and haplogroup determination

The complete mtDNA was amplified from total DNA in 24 overlapping 800–1,000-bp-long polymerase chain reaction (PCR) fragments. The PCR fragments were sequenced on both strands in an ABI 3710 sequencer (Applied Biosystems, Foster City, CA). Assembly and identification of variants in the mtDNA was carried out using the Staden Package (Staden et al. 2000). Using all identified mtDNA sequence variations, the samples were classified into mtDNA haplogroups.

### Quantification of mtDNA in cybrid cell lines

mtDNA content was measured by a multiplex real-time PCR method using the Applied Biosystems StepOnePlus PCR System (Applied Biosystems) as previously described (Andreu et al. 2009). Calibration curves were used to quantify mtDNA and nDNA copy number.

### Respiratory function assays

The rate of oxygen consumption was measured as described (Hofhaus et al. 1996) with slight modifications. Aliquots of 1.5 × 10^6^ cells were resuspended in 300 *μ*L of DMEM without glucose, supplemented with pyruvate and glutamine, the rate of O_2_ consumption of whole cells was recorded for 3 min using a Clark O_2_ electrode (Hansatech Instruments, King's Lynn, Norfolk, U.K.). To assess respiratory chain enzymes activities in cells, 1.5 × 10^7^ cells were harvested by trypsinization, washed twice with phosphate buffer, resuspended in 1 mL of SETH buffer (250 mmol/L sucrose, 2 mmol/L EDTA, 10 mmol/L Tris-HCl pH = 8, 100 U/L Heparin, pH = 7.4) and sonicated in ice (three cycles, 10 sec, 15 *μ*m). The activities of rotenone-sensitive NADH-coenzyme Q1 reductase (complex I), succinate dehydrogenase (complex II), antimycin-sensitive ubiquinol cytochrome *c* reductase (complex III), cytochrome *c* oxidase (complex IV), and citrate synthase were measured as described (DiMauro et al. 1987).

Rate of ATP synthesis was assayed as described (Manfredi et al. 2001 using the ATP Bioluminescent Assay Kit (Sigma Aldrich, St. Louis, MO).

Mitochondrial membrane potential (Δ*Ψ*_M_) was assessed using the lipophilic cationic membrane potential-sensitive dye JC-1 (5,5′,6,6′-tetrachloro-1,1′,3,3′-tetraethylbenzimidazolylcarbocyanine iodide; Molecular Probes, Life Technologies, Carlsbad, CA) following manufacturer instruction and using a FacScan cytometer and Cell Quest software (Becton Dickinson, Franklin Lakes, NJ).

### Lactate measurements

One milliliter aliquots of medium were deproteinized with 73 *μ*L of 60% perchloric acid. The samples were centrifuged (14,000 *g*, 5 min), and supernatant were neutralized using 1 mol/L KOH, and centrifuged again as before. Lactate levels in the supernatant were measured spectrophotometrically Enzychrom TM Lactate Kit Assay (BioAssay Systems, Hayward, CA).

### Reactive oxygen species production

Mitochondrial superoxide was assessed using the fluorogenic dye MitoSOX (Molecular Probes) following manufacturer's instructions and analyzed by flow cytometry using FacScan and Cell Quest software (Becton Dickinson).

Cellular hydrogen peroxide production was measured using 5-(and-6)-carboxy-2′,7′-dichlorodihydrofluorescein diacetate (carboxy-H_2_DCFDA, Molecular Probes) following manufacturer instruction and analyzed by flow cytometry using FacScan and Cell Quest software (Becton Dickinson).

### Modified pathogenicity scoring system

Table 1 shows proposed modified scoring system including the new score for mutations with normal mitochondrial activity in functional studies.

**Table 1:** The pathogenic scoring system.

Scoring criteria	Score/20	
More than one independent report	Yes	2
	No	0
Evolutionay conservation of the base or base-pair	One change	2
	Two changes	1
	Multiple changes	0
Variant heteroplasmy	Yes	2
	No	0
Segregation of the mutation with disease	Yes	2
	No	0
Histochemical evidence of mitochondrial disease	Strong evidence	2
	Weak evidence	1
	No evidence	0
Biochemical defect in complexes I, III or IV	Yes	2
	No	0
Evidence of mutation segregation with biochemical defect from single-fiber studies	Yes	3
	No	0
Mutant mt-tRNA steady-state level studies or evidence of pathogenicity in *trans*-mitochondrial cybrid studies	Yes	5
	No	0
Evidence of normality in *trans*-mitochondrial cybrid studies	Yes	−5
	No	0
Thresholds for the scoring system		
≤6 points	Neutral polymorphism		
7–10 points	Possibly pathogenic		
11–13 points (not included evidence from single-fiber, steady-state level, or *trans*-mitochondrial cybrid studies)	Probably pathogenic		
≥11 points (including evidence from single fiber, steady-state level or *trans*-mitochondrial cybrid studies)	Definitely pathogenic		

The proposal to update the original pathogenicity scoring system (Yarham et al. 2011) including a new negative score for mutations showing normal mitochondrial function in functional studies.

### Statistic

Mann–Whitney *U-*test or Student *t*-test was used to compare the results obtained in the functional characterization of the cybrid cell lines.

## Results

Using the scoring system proposed by Yarham et al. the m.8296A>G mutation was classified as possibly pathogenic (10 points derived from: >1 report, heteroplasmy, conservation, segregation with disease, and histochemistry) while the m.8347A>G was classified as a neutral polymorphism (4 points derived from: heteroplasmy and segregation with disease) (Yarham et al. 2011). Both changes obtained 0 points in the section focused on functional studies.

m.8296A>G has been associated with different phenotypes including diabetes (Kameoka et al. 1998), MELAS (Sakuta et al. 2002), MERFF (Arenas et al. 1999), stroke (Finnila et al. 2001), and hypertrophic cardiomyopathy (Akita et al. 2000). Despite the demonstration of the neutral effect of this mutation on the mitochondrial function, m.8296A>G continues to be reported as being associated with disease and it is classified as pathogenic in mitochondrial databases such as MITOMAP (Ruiz-Pesini et al. 2007) or Mamit-tRNA (Putz et al. 2007). The m.8347A>G mutation has been previously associated with myopathy (Brinckmann et al. 2007), essential hypertension (Zhu et al. 2009) and described as a variant associated with V3 haplogroup (Alvarez-Iglesias et al. 2009) but its effect on mitochondrial function has not been studied yet.

Previous studies of our group, performed using *trans*-mitochondrial cybrids derived from an African L1 haplogroup carrier, showed that m.8296A>G mutation is not pathogenic (Bornstein et al. 2002). However, the majority of the cases reported this mutation in the Japanese population, where L1 haplogroup is extremely infrequent. In order to rule out a haplogroup-specific effect, *trans*-mitochondrial cybrids containing the m.8296A>G mutation were generated on an Asiatic mitochondrial haplogroup B background (B4c1a1a). m.8347A>G cybrids were also derived from a B haplogroup (B4b1a1a) carrier and both cybrid lines were compared with a cybrid line derived from a healthy B haplogroup (B4b1a1) carrier. To ensure that the mtDNA levels had been restored after the cybridization process, mtDNA levels were in all cases determined until obtaining steady-state levels. When comparing the mtDNA levels of mutant and WT cybrids, we did not observe any significant differences (data not shown). Furthermore, the presence of additional non polymorphic changes was evaluated and ruled out by sequencing the complete mitochondrial genomes of all the cybrid cell lines (Table S1).

Table 2 shows the results of the OXPHOS function assays for both cybrid lines and the parental lines, 143B and 143B *ρ*^0^, compared with the haplogroup B-matched control line (Control B). Cybrid lines harboring the m.8347A>G mutation showed a dramatic deficiency in the OXPHOS system. The activities of complex I, III, and IV were significantly diminished compared with the control B, causing a clear defect in mitochondrial ATP synthesis (4% of control B) and oxygen consumption (24% of the control) rates. This is the first biochemical evidence of the pathogenicity of the m.8347A>G mutation. On the other hand, the m.8296A>G mutation on a mitochondrial background corresponding to haplogroup B did not cause any mitochondrial dysfunction, as previously described with the same mutation on a haplogroup L1 background.

**Table 2:** Rate of oxygen consumption, mitochondrial ATP synthesis and activities of the respiratory chain complexes.

	OXPHOS activity		Specific enzyme activity			
Cell line	O_2_ consumption	ATP synthesis	Complex I	Complex II	Complex III	Complex IV
143B	4.60 ± 1.29	1.10 ± 0.41	4.56 ± 0.44	6.26 ± 1.83	62.28 ± 7.91	37.56 ± 10.76
143B *ρ*^0^	0.17 ± 0.05	0.03 ± 0.02*	1.94 ± 0.72*	2.82 ± 0.49	7.37 ± 8.65***	3.25 ± 1.06***
m.8347A>G	0.60 ± 0.20	0.04 ± 0.03**	2.09 ± 1.18*	3.59 ± 0.44	7.55 ± 6.66***	5.00 ± 2.07**
m.8296A>G	3.60 ± 0.97	1.01 ± 0.33	4.70 ± 0.67	7.00 ± 0.29	51.45 ± 7.70	31.65 ± 8.89
Control B	2.53 ± 1.00	1.12 ± 0.41	5.88 ± 0.69	5.74 ± 0.42	66.06 ± 10.68	31.84 ± 5.84
*n*	3	5	5	5	5	5

Results obtained comparing the results of each cell line with the control B cell line results for each parameter.

* *P* < 0.05,

** *P* < 0.01,

*** *P* < 0.001 (Mann–Whitney *U-*test).

**Figure 1:**
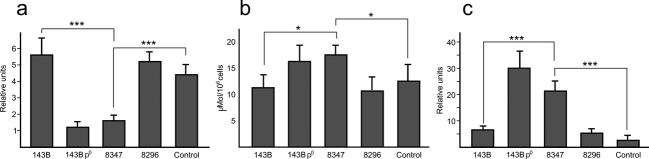
Mitochondrial membrane potentials, lactate production, and H_2_O_2_ production in the cell lines used in this study. (A) Mitochondrial membrane potential (*n* = 4). (B) Lactate production (*n* = 4). (c) H_2_O_2_ production (*n* = 4) Student's *t*-test, *P < 0.05, ***P < 0.001 8347: cybrid cell line carrying the m8347A>G mutation. 8296: cybrid cell line carrying the m8296A>G mutation. Control: cybrid cell line carrying a haplogroup B-matched mtDNA obtained from a healthy donor.

The mitochondrial alterations shown in m.8347A>G carriers, and their absence in m.8296A>G carriers, were reinforced by measuring mitochondrial membrane potentials (Δ*Ψ*_M_). Figure 1a shows a clear decrease in mitochondrial membrane potential in cybrid lines harboring the m.8347A>G mutation compared with control B (*P* < 0.001). Cybrid lines harboring m.8296A>G mutation showed a normal mitochondrial membrane potential, similar to the control B line.

To evaluate the metabolic state in the cybrid lines we measured the lactate levels in the culture media. In agreement with the results on OXPHOS function, the m.8347A>G harboring cell line showed a 55% increase in lactate production when compared with control (*P* < 0.05) and very similar to that found in *ρ*^0^ cells, which correlates well with an increase in the glycolytic flux as a compensatory mechanism for the severe defect in mitochondrial ATP synthesis (Fig. 1B). In agreement with their normal OXPHOS function, cybrid cell lines harboring m.8296A>G mutation showed normal lactate concentrations.

Mutations in mtDNA can produce an increase in reactive oxygen species (ROS) and this can play a major role in the pathogenic mechanisms of mitochondrial encephalomyopathies (Vives-Bauza et al. 2006). To test the possible role of oxidative stress in the biochemical phenotype of these mutations, mitochondrial superoxide and cellular hydrogen peroxide production were measured in all cell lines.

The mitochondrial electron transport chain is the main source of superoxide anion, a precursor of most other ROS (Turrens 2003). Cells lines harboring m.8296A>G or m.8347A>G mutations did not show any significant difference with control in superoxide anion production (data not shown). However, when the cellular H_2_O_2_ levels were measured a dramatic increase in m.8347A>G cell lines (*P* < 0.001) became evident, indicating that the cellular red-ox balance was altered in this cell line (Fig. 1C).

Taking into account the results of the cybrid studies, we reevaluated both mutations using the original scoring system and the data from Table 2. m.8347A>G mutation impairs severely the OXPHOS function and therefore, it can be classified as definitely pathogenic (11/20 points, including *trans*-mitochondrial studies). On the other, hand the results obtained from the leftacterization of m.8296A>G mutation show a normal mitochondrial function; the score of this mutation remains unaffected and it remains classified as possibly pathogenic (10/20 points). However, when the proposed negative score was applied to m.8296A>G mutation, it scored 5/20 points, allowing its classification as neutral polymorphism.

## Discussion

A recent article published in *Human Mutation* by Yarham et al. (2011) reevaluated the pathogenicity scoring system proposed by the same group in 2004 to help to distinguish pathogenic from polymorphic changes in mitochondrial tRNA genes. This scoring system, based on the canonical criteria proposed by DiMauro and Schon (2001) for pathogenic mitochondrial tRNA mutations, gives a special weight to functional data, like those obtained from *trans*-mitochondrial cybrid studies. However, only mutations resulting in a defect in mitochondrial function are considered in this algorithm while mutations showing proper mitochondrial function are ignored. This difference in the weight assigned to positive and negative results is translated in differences in the robustness of the scoring system when it is used to confirm or rule out pathogenicity. Identifying disease-causing mutations is equally important as to classifying changes as neutral polymorphisms, as they are in fact two sides of the same coin. The latter type of classification avoids false mutation-disease associations which could lead to misdiagnosis.

Our results show that the m.8347A>G mutation severely impairs the OXPHOS function, and allow its reclassification as definitely pathogenic (11/20 points, including *trans*-mitochondrial studies), evidencing the utility of the cybrid model in assigning pathogenicity to changes in the mtDNA.

We also studied the m.8296A>G change, that affects a well-conserved nucleotide located in the acceptor stem of the mitochondrial tRNA^Lys^ and has been found both as a homoplasmic and as a heteroplasmic variant in several patients (Arenas et al. 1999; Akita et al. 2000). All findings together, that is evolutionary conservation, variant heteroplasmy, segregation of the mutation with the disease, and histochemical evidence of a mitochondrial defect summed a total of 10 points in the pathogenicity scoring system proposed by Yarham et al. (2011), resulting in a classification as possibly pathogenic. Our group reported in 2002 that this mutation does not cause mitochondrial dysfunction, using a *trans*-mitochondrial cybrid approach. In this case, the mutation was homoplasmic in a patient presenting a phenotype compatible with MERRF syndrome, and this patient also carried the m.8363G>A mutation (Arenas et al. 1999). Using the same cybrid model approach, we showed that this mutation induced a clear mitochondrial dysfunction and was therefore the real cause of the phenotype found in the patient (Bornstein et al. 2005). However, despite the demonstration of the neutral effect of this mutation on the mitochondrial function in this patient, m.8296A>G continues being reported associated to disease and considered as pathogenic (Putz et al. 2007; Ruiz-Pesini et al. 2007; Usami et al. 2012).

To exclude the possible effect of the mtDNA haplogroup, the m.8296A>G mutation was studied in a background of haplogroup B, and similar results to those described for this mutation on haplogroup L1 were found. However, the scoring system of Yarham et al. (2011) classifies it as possibly pathogenic (10/20 points), despite the fact that the effect of this mutation on the OXPHOS system has been ruled out independently in two different mitochondrial backgrounds. The contrast in the application of the scoring system to these mutations shows the asymmetry between the weights assigned to positive and negative results of functional studies.

We entirely agree with the importance assigned to functional studies to determine the pathogenicity of mt-tRNA mutations in the scoring system described by Yarham et al. (2011). But at the same time, we think that the value of these studies should not be limited to confirming the pathogenicity of those mutations with positive evidence of a functional defect; negative results obtained in such studies should also be considered to rule out the pathogenicity of genetic changes that have a high score derived from other criteria. We consider the m.8296A>G nucleotide change as a good example of the benefits of taking into account negative results obtained in cybrid analysis to classify mtDNA nucleotide variants as pathogenic or polymorphic.

We think that the score for a nucleotide change which fails to show any mitochondrial defect when studied using one of the established methods to assess pathogenicity, like *trans*-mitochondrial cybrid studies, should not be the same as the score for a change that has not yet been studied. We therefore think that an additional criterion for these cases is appropriated. Thus, we propose a modification in the scoring system, introducing a new score for those cases in which the studies using *trans*-mitochondrial cybrids fail to show any defect in the OXPHOS system (Table 1). In our opinion, this modification will allow a more accurate discrimination between a pathogenic mutation and a neutral polymorphism.

In summary, we hope that our proposal to include a negative scoring for mutations which fail to show any mitochondrial defect in functional studies will help to improve the scoring system in order to facilitate the diagnosis of this complex group of diseases.

## Supporting Information

Table S1 mtDNA variations identified in the cybrid cell line containing the (A) m. 8347A>G change, (B) m. 8296A>G change, (C) mtDNA variations identified in a control cybrid cell line.
