# Supplementary material for: The pathogenicity scoring system for mitochondrial tRNA mutations revisited
Source: Mol Genet Genomic Med. 2013 Nov 11;2(2):107–14. doi: 10.1002/mgg3.47 (PMC3960052; doi:10.1002/mgg3.47)
Supplement: Table S1 — mtDNA variations identified in the cybrid cell line containing the (A) m. 8347A>G change, (B) m. 8296A>G change, (C) mtDNA variations identified in a control cybrid cell line. [file mgg30002-0107-sd1.doc]

# Supplementary Table 1

# A. mtDNA variations identified in the cybrid cell line containing the m. 8347A>G change

|  | **GENE** | **mtDNA sequence variations** |
| --- | --- | --- |
| **Cybrid line 1** | **MT-RNR1** | m.750A>G, m.827A>G, m.1438A>G |
|  | **MT-RNR2** | m.2706A>G, m.2831G>A |
|  | **MT-ND1** | m.4117T>C p.(=); GenBank p.MT-ND1: NP_536843.1 |
|  | **MT-ND2** | m.4769A>G p.(=), m.4820G>A p.(=); GenBank p.MT-ND2: NP_536844.1 |
|  | **MT-CO1** | m.6023G>A p.(=), m.6413T>C p.(=), m.7028C>T p.(=); GenBank p.MT-CO1: NP_536845.1 |
|  | **MT-CO2** | m.7664G>A p.(Ala27Thr), m.8206G>A p.(=); GenBank p.MT-CO2: NP_536846.1 |
|  | **MT-NC7** | m.8281_8289delCCCCCTCTA |
|  | **MT-TK** | m.8347A>G |
|  | **MT-ATP6** | m.8860A>G p.(Thr112Ala); GenBank p.MT-ATP6: NP_536848.1 |
|  | **MT-ND4** | m.11719G>A p.(=); GenBank p.MT-ND4: NP_536852.1 |
|  | **MT-ND5** | m.13590G>A p.(=); GenBank p.MT-ND5: NP_536853.1 |
|  | **MT-CYB** | m.14766C>T p.(Ile7Thr), m.15236A>G p.(Ile164Val), m.15326A>G p.(Thr194Ala), m.15535C>T p.(=); GenBank p.MT-CYB: NP_536855.1 |
|  | **MT-DLOOP** | m.73A>G, m.199T>C, m.202A>G, m.207G>A, m.263A>G, m.302_303insC, m.310_311insC, m.499G>A, m.16030C>T, m.16136T>C, m.16183A>C, m.16189T>C, m.16217T>C, m.16284A>G, m.16519T>C |

# B. mtDNA variations identified in the cybrid cell line containing the m. 8296A>G change

|  | **GENE** | **mtDNA sequence variations** |
| --- | --- | --- |
| **Cybrid line 2** | **MT-RNR1** | m.709G>A, m.750A>G, m.1119T>C, m.1438A>G |
|  | **MT-RNR2** | m.2706A>G, m.2831G>A |
|  | **MT-ND1** | m.3497C>T p.(Ala64Val) GenBank p.MT-ND1: NP_536843.1 |
|  | **MT-ND2** | m.4769A>G p.(=), m.5450C>T p.(=); GenBank p.MT-ND2: NP_536844.1 |
|  | **MT-CO1** | m. 7028C>T p.(=); GenBank p.MT-CO1: NP_536845.1 |
|  | **MT-NC7** | m.8281_8289delCCCCCTCTA |
|  | **MT-TK** | m.8296A>G |
|  | **MT-ATP6** | m.8860A>G p.(Thr112Ala), m.8950G>A p.(Val142Ile); GenBank p.MT-ATP6: NP_536848.1 |
|  | **MT-CO3:** | m.9479T>C p.(=); GenBank p.MT-CO3: NP_536849.1 |
|  | **MT-ND3** | m.10310G>A p.(=); GenBank p.MT-ND3: YP_003024033.1 |
|  | **MT-ND4** | m.11719G>A p.(=); GenBank p.MT-ND4: NP_536852.1 |
|  | **MT-ND5** | m.14133A>G p.(=); GenBank p.MT-ND5: NP_536853.1 |
|  | **MT-CYB** | m.14766C>T p.(Ile7Thr), m.15326A>G p.(Thr194Ala), m.15346G>A p.(=); GenBank p.MT-CYB: NP_536855.1 |
|  | **MT-DLOOP** | m.73A>G, m.263A>G, m.302_303insCC, m.310_311insC, m.16086T>C, m.16189T>C, m.16217T>C, m.16293A>G, m.16311T>C, m.16519T>C |

# C. mtDNA variations identified in a control cybrid cell line

|  | **GENE** | **mtDNA sequence variations** |
| --- | --- | --- |
| **Control cybrid line** | **MT-RNR1** | m.750A>G, m. 827A>G, m.1438A>G |
|  | **MT-RNR2** | m.2706A>G, m.2831G>A |
|  | **MT-ND1** | m.4117T>C p.(=), m.4216T>C p.(Tyr304His); GenBank p.MT-ND1: NP_536843.1 |
|  | **MT-ND2** | m.4769A>G p.(=), m.4820G>A p.(=); GenBank p.MT-ND2: NP_536844.1 |
|  | **MT-CO1** | m.6023G>A p.(=), m.6413T>C p.(=), m.7028C>T p.(=); GenBank p.MT-CO1: NP_536845.1 |
|  | **MT-CO2** | m.8206G>A p.(=); GenBank p.MT-CO2: NP_536846.1 |
|  | **MT-NC7** | m.8281_8289delCCCCCTCTA |
|  | **MT-ATP6** | m.8860A>G p.(Thr112Ala); GenBank p.MT-ATP6: NP_536848.1 |
|  | **MT-ND4** | m.11719G>A p.(=); GenBank p.MT-ND4: NP_536852.1 |
|  | **MT-ND5** | m.13590G>A p.(=); GenBank p.MT-ND5: NP_536853.1 |
|  | **MT-CYB** | m.14766C>T p.(Ile7Thr), m.15236A>G p.(Ile164Val), m.15326A>G p.(Thr194Ala), m.15535C>T p.(=); GenBank p.MT-CYB: NP_536855.1 |
|  | **MT-DLOOP** | m.73A>G, m.146T>C, m.199T>C, m.202A>G, m.207G>A, m.263A>G, m.302_303insC, m.310_311insC, m.499G>A, m.16136T>C, m.16217T>C, m.16284A>G, m.16519T>C |

**Footnote for Supplementary Table**. Reference sequence is the human mitochondrial DNA revised Cambridge sequence (<http://www.mitomap.org/mitoseq.html>). MT-RNR1: mitochondrially encoded 12S rRNA; MT-RNR2: mitochondrially encoded 16S rRNA; MT-ND1, MT-ND2, MT-ND3, MT-ND4, MT-ND5, MT-ND6: mitochondrially encoded NADH dehydrogenase subunits 1, 2, 3, 4, 5 and 6; MT-NC7: non coding nucleotides; MT-CO1,MT-CO2, MT-CO3: mitochondrially encoded cytochrome c oxidase I, II and III, MT-TK: mitochondrially encoded tRNA Lys; MT-CYB: mitochondrially encoded cytochrome b; MT-ATP6: mitochondrially encoded ATP synthase 6; MT-DLOOP: polymorphisms identified in the mtDNA control region.
